# Impact Mechanical Response of a 2-2 Cement-Based Piezoelectric Sensor Considering the Electrode Layer Effect

**DOI:** 10.3390/s17092035

**Published:** 2017-09-06

**Authors:** Taotao Zhang, Keping Zhang, Wende Liu, Yangchao Liao

**Affiliations:** 1 School of Transportation Science and Engineering, Beihang University, Beijing 100191, China; kepingzhang@buaa.edu.cn (K.Z.); liaoyc@buaa.edu.cn (Y.L.); 2 National Institute of Metrology, Beijing 100023, China; wendeliu@nim.ac.cn

**Keywords:** 2-2 cement-based piezoelectric sensor, impact response, theoretical solutions, electrode layer effect

## Abstract

Cement-based piezoelectric composite, has been widely used as a kind of smart material in structural health monitoring and active vibration control. However, transient dynamic loads such as impact loads may cause serious damage to the composite. Considering the electrode layer effect, this paper aimed to investigate the theoretical response of a 2-2 cement-based piezoelectric composite sensor subjected to an impact load. The vibration behaviors are analyzed by using the mode summation method and the virtual work principle. To simulate the impact load, transient haversine wave loads are assumed in the numerical simulation. Close agreements between theoretical and numerical solutions are found for peak transient haversine wave loads larger than 500 kPa, therefore proving the validity of the theory. Moreover, the influence of the electrode material and geometrical parameters on the dynamic characteristics of this sensor are considered. The present work should be beneficial to the design of this kind of sensor by taking into account the electrode layer effect.

## 1. Introduction

With the advances in smart or intelligent structure technology, piezoelectric composites are widely used in structural health monitoring [1,2,3]. These smart devices are sensitive to the influences from the external environment and have extraordinary compatibility with the most popular construction materials used in civil engineering such as cement and concrete. The design and fabrication of composite materials open new avenues for optimizing the electrical, magnetic, and mechanical properties of sensors for specific applications [4,5,6].

According to the different connectivity of each phase in the piezoelectric composite, piezoelectric composites can be divided into 10 basic types such as 0-0, 0-1, 0-3, 2-2 and so on. The first number represents the piezoelectric phase and the second number represents the non-piezoelectric phase. A 2-2 cement-based piezoelectric composite is the composite where a two-dimensional piezoelectric plate is embedded in a two-dimensional cement matrix [7]. Li and Zhang fabricated 2-2 cement-based piezoelectric composites using hardened Portland cement and studied the sensing performance of such composites at low frequency [8]. Investigation of the dielectric and acoustic impedance properties of 2-2 barium zirconate titanate-Portland cement composites reveals that the composites have higher piezoelectric voltage coefficients and lower acoustic impedance values than pure ceramic [9]. Cheng et al. studied the effects of composite thickness on the dielectric, piezoelectric and electromechanical properties of the composite [10]. The receiving piezoelectric transducer can be fabricated by decreasing the thickness. Moreover, mathematical models to describe the deformation and electrical behaviors have also been studied by numerous researchers. For example, based on the theory of elasticity, static analyses of piezoelectric curved composites and multi-layered piezoelectric cantilevers were performed by Zhang et al. [11,12,13,14], who further established an analytical model of the dynamic properties of the 2-2 cement-based piezoelectric transducer subjected to a uniformly distributed harmonic load and external harmonic electrical potential [15]. Chen et al. analyzed the free vibration of laminates beams by using the state space method and the differential quadrature method [16]. Wang and Shi presented the dynamic behaviors of piezoelectric composite stack transducers and discussed the influence of the electrode thickness on their dynamic characteristics [17].

Few of the published papers have studied the dynamic performance of piezoelectric composite sensors subjected to impact loads, especially the theoretical aspect. Zhang et al. studied the dynamic properties of piezoelectric structures subjected to simpact load and gave theoretical solutions of the mechanical and electrical behaviors of the piezoelectric structures [18]. Yin et al. presented a mixed finite element formulation for modelling the behavior of a piezoelectric composite and analyzed the response of distributed sensors made of PVDF film when the composite was subjected to low velocity impacts [19]. Ueda studied the transient response of a functionally graded piezoelectric material strip with a vertical crack under the action of normal impacts [20,21]. In many engineering applications, piezoelectric composite may experience transient dynamic loads, which could induce certain hidden damages (matrix cracking, fiber breakage, etc.). It is therefore important to understand the dynamic behaviors of cement-based piezoelectric sensors.

In the present study, we aim to provide a mathematical model to describe the impact mechanical response of a 2-2 cement-based piezoelectric composite subjected to impact load. The fundamental equations and wave equations are summarized in Section 2 based upon piezo-elasticity. In the following section, the vibration behaviors of the composite are obtained by using the mode summation method and the principle of virtual work. In Section 4, the transient harversine wave load is used for the numerical simulation. The numerical results verified the analytical solutions. Moreover, the influence of the material and geometrical parameters on mechanical and electrical behaviors of sensor is discussed. It can be seen that the proposed model would provide guidance for sensor structure design, material selection and impact load design in simulations and experiments.

## 2. Basic Equations

**Figure 1:**
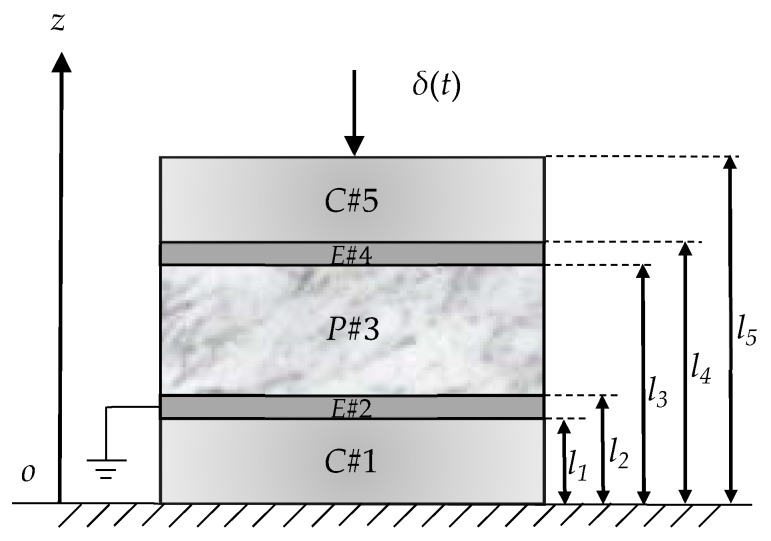
Schematic 2-2 cement-based piezoelectric sensor under impact load.

The 2-2 cement-based piezoelectric sensor consists of a piezoelectric layer P#3 , elastic electrode layers E#2 , E#4 and cement layers C#1 , C#5 , which are arranged in an alternating manner as shown in Figure 1. 

The thickness of layer i are determined by $l_{i+1}-l_{i}$ ( i = 1, 2, 3, 4, 5 ). The composite is excited longitudinally by the impact load δ(t) at its free end. The piezoelectric layer is polarized along its thickness direction and the electrode surface is perpendicular to the thickness direction of the piezoelectric layer. In order to analyze the transient phase of the composite vibration, the following assumptions are adopted: (1) all of the constitutive materials are linear and the deformations are small; (2) one-dimensional assumption. When the composite is deformed, the cross-section maintain a plane and the axial stress is uniformly distributed on the cross-section; (3) damping is not considered. For the piezoelectric layer P#3 , the body force and body charge are neglected, the governing equations can be expressed as follows:

(1) $$\{\begin{array}{c} \frac{\partial^{2}w_{p3}}{\partial t^{2}}=\frac{1}{\rho_{p}}\frac{\partial \sigma_{p3}}{\partial z}\\ \sigma_{p3}=C_{33p}\varepsilon_{p3}-e_{33}E_{z}\\ \varepsilon_{p3}=\frac{\partial w_{p3}}{\partial z} \end{array}$$

(2) $$\{\begin{array}{c} D_{z}=e_{33}\varepsilon_{p3}+\varepsilon_{33}^{S}E_{z}\\ E_{z}=-\frac{\partial \phi }{\partial z}\\ \frac{\partial D_{z}}{\partial z}=0 \end{array}$$

where $\sigma_{p3}$ , $\varepsilon_{p3}$ and $w_{p3}$ are the stress, strain, and displacement of the piezoelectric layer P#3 along *z*-direction, respectively. $D_{z}$ and φ represent the electric displacement and electric potential in the along *z*-direction. $C_{33p},\;e_{33}$ and $\varepsilon_{33}^{S}$ are the elastic stiffness coefficient, piezoelectric coefficient and permittivity coefficient of the piezoelectric material, respectively. $\rho_{p}$ is the density of the piezoelectric material. Combining Equations (1) and (2), the following equation are obtained:

(3) $$\{\begin{array}{c} \rho_{p}\frac{\partial^{2}w_{p3}}{\partial t^{2}}=C_{33p}\frac{\partial^{2}w_{p3}}{\partial z^{2}}+e_{33}\frac{\partial^{2}\phi }{\partial z^{2}}\\ \varepsilon_{33}^{S}\frac{\partial^{2}\phi }{\partial z^{2}}=e_{33}\frac{\partial^{2}w_{p3}}{\partial z^{2}} \end{array}$$

Combining Equation (3), one gets the following wave equation:

(4) $$\frac{\partial^{2}w_{p3}}{\partial t^{2}}-C_{a}{}^{2}\frac{\partial^{2}w_{p3}}{\partial z^{2}}=0$$

where $C_{a}\;=\sqrt{E_{0}/\rho_{p}}\;$ , $E_{0}{\;=\;C}_{33p}{\;+\;e}_{33}{}^{2}/\varepsilon_{33}^{S}$ . $C_{a}$ represents the propagation velocity of the vibration wave in the piezoelectric material.

For the cement layer C#i (i = 1, 5) , the body charge are neglected, and the basic equations can be expressed as follow:

(5) $$\{\begin{array}{c} \frac{\partial^{2}w_{ci}}{\partial t^{2}}=\frac{1}{\rho_{c}}\frac{\partial \sigma_{ci}}{\partial z}\\ \sigma_{ci}=C_{33c}\varepsilon_{ci}\\ \varepsilon_{ci}=\frac{\partial w_{ci}}{\partial z} \end{array}$$

where $\sigma_{ci}$ , $\varepsilon_{ci}$ and $w_{ci}$ are the stress, strain, and displacement of the cement layer C#i along *z*-direction, respectively. $C_{33c}$ and $\rho_{c}$ are the elastic stiffness coefficient and density of the cement material. Combining Equation (5), one gets the following wave equation:

(6) $$\frac{\partial^{2}w_{ci}}{\partial t^{2}}-C_{b}{}^{2}\frac{\partial^{2}w_{ci}}{\partial z^{2}}=0$$

where $C_{b}\;=\;\sqrt{C_{33c}∕\rho_{c}}$ and represents the propagation velocity of the vibration wave in the cement material.

For the elastic electrode layer E#i (i = 2, 4), the body charge are neglected, and the basic equations can be expressed as follow:

(7) $$\{\begin{array}{c} \frac{\partial^{2}w_{Ei}}{\partial t^{2}}=\frac{1}{\rho_{E}}\frac{\partial \sigma_{Ei}}{\partial z}\\ \sigma_{Ei}=C_{33E}\varepsilon_{Ei}\\ \varepsilon_{Ei}=\frac{\partial w_{Ei}}{\partial z} \end{array}$$

where $\sigma_{Ei}$ , $\varepsilon_{Ei}$ and $w_{Ei}$ are the stress, strain, and displacement of the elastic electrode layer E#i along *z*-direction, respectively. $C_{33E}$ and $\rho_{E}$ are the elastic stiffness coefficient and density of the elastic electrode material. Similarly, wave equation can be easily obtained:

(8) $$\frac{\partial^{2}w_{Ei}}{\partial t^{2}}-C_{c}{}^{2}\frac{\partial^{2}w_{Ei}}{\partial z^{2}}=0$$

where $C_{c}\;=\;\sqrt{C_{33E}∕\rho_{E}}$ and represents the propagation velocity of the vibration wave in the elastic electrode material.

## 3. Accurate Vibration Analysis of 2-2 Cement Based Piezoelectric Composite

Figure 1 shows the 2-2 cement-based piezoelectric composite sensor subjected to the impact load. In order to obtain the accurate vibration solution, we use the mode summation method and the principle of the virtual work in this section. It is assumed that the solutions for the displacements are:

(9) $$\{\begin{array}{c} w_{pi}(z,t)=Z_{pi}(z)T_{n}(t);\;i=3\\ w_{ci}(z,t)=Z_{ci}(z)T_{n}(t);\;i=1,5\\ w_{Ei}(z,t)=Z_{Ei}(z)T_{n}(t);\;i=2,4 \end{array}$$

where $Z_{ci}\left(z\right)$ , $Z_{Ei}\left(z\right)$ and $Z_{pi}\left(z\right)$ are normal modes of the cement, piezoelectric and elastic electrode layers for the longitudinal vibration. $T_{n}$ is the function of *t* which can be determined by the initial conditions.

Substituting the above equations into Equations (4), (6) and (8), we obtain:

(10) $$\{\begin{array}{c} Z_{pi}{}^{\prime \prime }(z)+\lambda_{i}Z_{pi}(z)=0;\;l_{i-1}\leq z\leq l_{i},\;i=3\\ Z_{ci}{}^{\prime \prime }(z)+\lambda_{i}Z_{ci}(z)=0;\;l_{i-1}\leq z\leq l_{i},\;i=1,5\\ Z_{Ei}{}^{\prime \prime }(z)+\lambda_{i}Z_{Ei}(z)=0;\;l_{i-1}\leq z\leq l_{i},\;i=2,4 \end{array}$$

(11) $$\{\begin{array}{c} T_{n}{}^{\prime \prime }(t)+\lambda_{i}C_{a}{}^{2}T_{n}(t)=0;\;t\geq 0,\;i=3\\ T_{n}{}^{\prime \prime }(t)+\lambda_{i}C_{b}{}^{2}T_{n}(t)=0;\;t\geq 0,\;i=1,5\\ T_{n}{}^{\prime \prime }(t)+\lambda_{i}C_{c}{}^{2}T_{n}(t)=0;\;t\geq 0,\;i=2,4\; \end{array}$$

where $\lambda_{i}$ are eigenvalues.

By solving Equation (10), the solutions of the normal modes are shown as follow:

(12) $$\{\begin{array}{c} Z_{pi}(z)=a_{in}\cos\sqrt{\lambda_{in}}z+b_{in}\sin\sqrt{\lambda_{in}}z;\;l_{i-1}\leq z\leq l_{i},\;i=3\\ Z_{ci}(z)=a_{in}\cos\sqrt{\lambda_{in}}z+b_{in}\sin\sqrt{\lambda_{in}}z;\;l_{i-1}\leq z\leq l_{i},\;i=1,5\\ Z_{Ei}(z)=a_{in}\cos\sqrt{\lambda_{in}}z+b_{in}\sin\sqrt{\lambda_{in}}z;\;l_{i-1}\leq z\leq l_{i},\;i=2,4 \end{array}$$

where $a_{in}$ and $b_{in}$ will be determined by the boundary conditions.

In order to solve $a_{in}$ and $b_{in}$ , the boundary and connecting conditions are written as (including the electrical boundary conditions and the initial conditions):

{(13a)wc1(0,t)=0;∂wc5(l5,t)∂z=0; t≥0(13b)wc1(l1,t)=wE2(l1,t);  wE2(l2,t)=wp3(l2,t);(13c)wp3(l3,t)=wE4(l3,t);  wE4(l4,t)=wc5(l4,t); t≥0(13d)C33c∂wc1(l1,t)∂z=C33E∂wE2(l1,t)∂z;  C33E∂wE2(l2,t)∂z=E0∂wp3(l2,t)∂z;(13e)E0∂wp3(l3,t)∂z=C33E∂wE4(l3,t)∂z;  C33E∂wE4(l4,t)∂z=C33c∂wc5(l5,t)∂z;  t≥0(13f)wpi(z,0)=∂wpi(z,0)∂t=0;  i=3(13g)wci(z,0)=∂wci(z,0)∂t=0;  i=1,5(13h)wEi(z,0)=∂wEi(z,0)∂t=0;  i=2,4(13i)ϕi|z=l2=0;D≡0

Substitution of Equation (9) into Equation (13) leads to:

{(14a)Zc1(l1)=ZE2(l2);  C33cZc1′(l1)=C33EZE2′(l1)(14b)ZE2(l2)=Zp3(l2);  C33EZE2′(l2)=E0Zp3′(l2)(14c)Zp3(l3)=ZE4(l3);  E0Zp3′(l3)=C33EZE4′(l3)(14d)ZE4(l4)=Zc5(l4);  C33EZE4′(l4)=C33cZc5′(l4)(14e)Zc1(0)=0;  Zc5′(l5)=0

By combining Equations (11), (13b,c) and (14a–c), one obtains the following relation:

(15) $$\sqrt{\lambda_{1}}C_{b}=\sqrt{\lambda_{2}}C_{c}=\sqrt{\lambda_{3}}C_{a}=\sqrt{\lambda_{4}}C_{c}=\sqrt{\lambda_{5}}C_{b}$$

Substitution of Equation (12) into Equation (14) leads to the following equations:

{(16a)a1n=0(16b)aincosλinli+binsinλinli−ai+1,ncosλi+1,nli−bi+1,nsinλi+1nli=0(16c)Ciλin(−ainsinλinli+bincosλinli)−Ci+1λi+1,n(−ai+1,nsinλi+1,nli+bi+1,ncosλi+1,nli)=0(16d)−a5nλ5nsinλ5nl5+b5nλ5ncosλ5nl5=0

here  i= 2, 3, 4, and correspondingly $C_{1}{\;=\;C}_{5}{\;=\;C}_{33c}{,\;C}_{2}{\;=\;C}_{4}{\;=\;C}_{33E}{,\;C}_{3}{\;=\;E}_{0}$ .

By solving the linear Equation (16b,c) with two unknowns, the expressions of $a_{in}$ and $b_{in}$ are obtained:

(17) $$\{\begin{array}{c} \begin{array}{c} a_{in}=a_{i-1,n}(\cos\sqrt{\lambda_{in}}l_{i-1}\cos\sqrt{\lambda_{i-1,n}}l_{i-1}+\frac{C_{i-1}\sqrt{\lambda_{i-1,n}}}{C_{i}\sqrt{\lambda_{in}}}\sin\sqrt{\lambda_{in}}l_{i-1}\sin\sqrt{\lambda_{in}}l_{i})+\\ \;b_{i-1,n}(\sin\sqrt{\lambda_{i-1,n}}l_{i-1}\cos\sqrt{\lambda_{in}}l_{i-1}-\frac{C_{i-1}\sqrt{\lambda_{i-1,n}}}{C_{i}\sqrt{\lambda_{in}}}\cos\sqrt{\lambda_{i-1,n}}l_{i-1}\sin\sqrt{\lambda_{in}}l_{i-1}) \end{array}\\ \begin{array}{c} b_{in}=a_{i-1,n}(\cos\sqrt{\lambda_{i-1,n}}l_{i-1}\sin\sqrt{\lambda_{in}}l_{i-1}-\frac{C_{i-1}\sqrt{\lambda_{i-1,n}}}{C_{i}\sqrt{\lambda_{in}}}\sin\sqrt{\lambda_{i-1,n}}l_{i-1}\cos\sqrt{\lambda_{in}}l_{i-1})+\\ \;b_{i-1,n}(\sin\sqrt{\lambda_{in}}l_{i-1}\sin\sqrt{\lambda_{i-1,n}}l_{i-1}+\frac{C_{i-1}\sqrt{\lambda_{i-1,n}}}{C_{i}\sqrt{\lambda_{in}}}\cos\sqrt{\lambda_{i-1,n}}l_{i-1}\cos\sqrt{\lambda_{in}}l_{i-1}) \end{array} \end{array}$$

here i=  2, 3, 4 and we assume that $b_{1n}\;=\;1$ .

It should be noted that when i=  4, there are three linear Equation (16b–d) with two unknowns:

(18) $$\{\begin{array}{c} a_{5n}\cos\sqrt{\lambda_{5n}}l_{4}+b_{5n}\sin\sqrt{\lambda_{5n}}l_{4}=a_{4n}\cos\sqrt{\lambda_{4n}}l_{4}+b_{4n}\sin\sqrt{\lambda_{4n}}l_{4}\\ a_{5n}\sin\sqrt{\lambda_{5n}}l_{4}-b_{5n}\cos\sqrt{\lambda_{5n}}l_{4}=\frac{C_{33E}\sqrt{\lambda_{4n}}}{C_{33C}\sqrt{\lambda_{5n}}}(a_{4n}\sin\sqrt{\lambda_{4n}}l_{4}-b_{4n}\cos\sqrt{\lambda_{4n}}l_{4})\\ -a_{5n}\sqrt{\lambda_{5n}}\sin\sqrt{\lambda_{5n}}l_{5}+b_{5n}\sqrt{\lambda_{5n}}\cos\sqrt{\lambda_{5n}}l_{5}=0 \end{array}$$

Nonhomogeneous equations have nontrivial solutions, which requires:

(19) $$\left|\begin{array}{ccc} \cos\sqrt{\lambda_{5n}}l_{4} & \sin\sqrt{\lambda_{5n}}l_{4} & a_{4n}\cos\sqrt{\lambda_{4n}}l_{4}+b_{4n}\sin\sqrt{\lambda_{4n}}l_{4}\\ \sin\sqrt{\lambda_{5n}}l_{4} & -\cos\sqrt{\lambda_{5n}}l_{4} & \frac{C_{33E}\sqrt{\lambda_{4n}}}{C_{33c}\sqrt{\lambda_{5n}}}(a_{4n}\sin\sqrt{\lambda_{4n}}l_{4}-b_{4n}\cos\sqrt{\lambda_{4n}}l_{4})\\ -\sin\sqrt{\lambda_{5n}}l_{5} & \cos\sqrt{\lambda_{5n}}l_{5} & 0 \end{array}\right|=0$$

By solving the above determinant, we obtain the following equation:

(20) $$\begin{array}{c} a_{4n}(\frac{C_{33E}\sqrt{\lambda_{4n}}}{C_{33c}\sqrt{\lambda_{5n}}}\sin\sqrt{\lambda_{4n}}l_{4}\cos\sqrt{\lambda_{5n}}h_{5}+\cos\sqrt{\lambda_{4n}}l_{4}\sin\sqrt{\lambda_{5n}}h_{5})+\\ b_{4n}(-\frac{C_{33E}\sqrt{\lambda_{4n}}}{C_{33c}\sqrt{\lambda_{5n}}}\cos\sqrt{\lambda_{4n}}l_{4}\cos\sqrt{\lambda_{5n}}h_{5}+\sin\sqrt{\lambda_{4n}}l_{4}\sin\sqrt{\lambda_{5n}}h_{5})=0 \end{array}$$

where $h_{5}{\;=\;l}_{5}-l_{4}$ .

Based on Equation (15), we define:

(21a)λ1nCb=λ2nCc=λ3nCa=λ4nCc=λ5nCb=λn¯(21b)h1=l1,h2=l2−l1,h3=l3−l2,h4=l4−l3,h5=l5−l4(21c)t1=l1Cb，t2=h2Cc，t3=h3Ca，t4=h4Cc,t5=h5Cb,T0=t1+t2+t3+t4+t5，  λn¯T0=λn¯¯

By combining Equation (21), one obtains:

(22) $$\sqrt{\lambda_{1n}}=\sqrt{\lambda_{5n}}=\frac{\sqrt{\bar{\bar{\lambda_{n}}}}}{A},\sqrt{\lambda_{2n}}=\sqrt{\lambda_{4n}}=\frac{\sqrt{\bar{\bar{\lambda_{n}}}}}{B},\sqrt{\lambda_{3n}}=\frac{\sqrt{\bar{\bar{\lambda_{n}}}}}{C}$$

where ${A\;=\;T}_{0}C_{b}$ , ${\;B\;=\;T}_{0}C_{c}$ , ${C\;=\;T}_{0}C_{a}$ .

By substituting Equation (22) into Equation (20), the value of $\sqrt{\bar{\bar{\lambda_{n}}}}$ is given by:

(23) $$\begin{array}{c} a_{4n}(\frac{A\cdot C_{33E}}{B\cdot C_{33c}}\sin\frac{\sqrt{\bar{\bar{\lambda_{n}}}}}{B}l_{4}\cos\frac{\sqrt{\bar{\bar{\lambda_{n}}}}}{A}h_{5}+\cos\frac{\sqrt{\bar{\bar{\lambda_{n}}}}}{B}l_{4}\sin\frac{\sqrt{\bar{\bar{\lambda_{n}}}}}{A}h_{5})+\\ b_{4n}(-\frac{A\cdot C_{33E}}{B\cdot C_{33c}}\cos\frac{\sqrt{\bar{\bar{\lambda_{n}}}}}{B}l_{4}\cos\frac{\sqrt{\bar{\bar{\lambda_{n}}}}}{A}h_{5}+\sin\frac{\sqrt{\bar{\bar{\lambda_{n}}}}}{B}l_{4}\sin\frac{\sqrt{\bar{\bar{\lambda_{n}}}}}{A}h_{5})=0 \end{array}$$

After we obtain the value of $\sqrt{\bar{\bar{\lambda_{n}}}}$ by Equation (23), $\sqrt{\lambda_{1n}}$ , $\sqrt{\lambda_{2n}}$ , $\sqrt{\lambda_{3n}}$ , $\;\sqrt{\lambda_{4n}}$ , and $\sqrt{\lambda_{5n}}$ can be obtained by Equation (21a).

To solve the expressions of $T_{n}\left(t\right)$ in Equation (9), we consider the initial condition of the sensor. By substituting Equation (9) into Equation (13f–h), we have:

(24) $$T_{n}\left(t\right)={\dot{T}}_{n}\left(t\right)=0$$

At t = 0 , there are three forces, namely the inertia forces, the elasticity force due to the deformation in each element of the composite, and the impact force δ(t) loaded at the free end. By taking any displacement δu satisfying the boundary and connecting conditions as a virtual displacement, according to Equation (12), δu can be given as follows:

{(25a)δupi=Zpi(z)=aincosλinz+binsinλinz; li−1≤z≤li,  i=3(25b)δuci=Zci(z)=aincosλinz+binsinλinz; li−1≤z≤li,  i=1,5(25c)δuEi=ZEi(z)=aincosλinz+binsinλinz; li−1≤z≤li,  i=2,4

The virtual work ${\delta W}_{i}$ of the inertia force on the virtual displacement is expressed as follows:

(26) $$\begin{array}{cc} \delta W_{i} & =\int_{0}^{l_{1}}\left(-\rho_{c}Adx\right)\cdot \frac{\partial^{2}w_{c1}}{\partial t^{2}}\cdot \delta u_{c1}+\int_{l_{2}}^{l_{2}}\left(-\rho_{E}Adx\right)\cdot \frac{\partial^{2}w_{E2}}{\partial t^{2}}\cdot \delta u_{E2}+\int_{l_{2}}^{l_{3}}\left(-\rho_{p}Adx\right)\cdot \frac{\partial^{2}w_{p3}}{\partial t^{2}}\cdot \delta u_{p3}\\ & +\int_{l_{3}}^{l_{4}}\left(-\rho_{E}Adx\right)\cdot \frac{\partial^{2}w_{E4}}{\partial t^{2}}\cdot \delta u_{E4}+\int_{l_{4}}^{l_{5}}\left(-\rho_{c}Adx\right)\cdot \frac{\partial^{2}w_{c5}}{\partial t^{2}}\cdot \delta u_{c5} \end{array}$$

By substituting Equations (9) and (25) into Equation (26), ${\delta W}_{i}$ can be obtained as:

(27) $$\delta W_{i}=-A{\ddot{T}}_{n}\left(t\right)\cdot \left(\rho_{c}B_{1}+\rho_{E}B_{2}+\rho_{p}B_{3}+\rho_{E}B_{4}+\rho_{c}B_{5}\right)$$

where $B_{i}\;=\;\int_{l_{i-1}}^{l_{i}}{\left(a_{in}\cos\sqrt{\lambda_{in}}{z\;+\;b}_{in}\sin\sqrt{\lambda_{in}}z\right)}^{2}dz$ , i=  1, 2, 3, 4, 5.

The virtual work ${\delta W}_{E}$ of the elastic force on the virtual displacement is expressed as follows:

(28) $$\begin{array}{cc} \delta W_{E} & =\int_{0}^{l_{1}}\left(C_{33c}Adx\right)\cdot \frac{\partial^{2}w_{c1}}{\partial z^{2}}\cdot \delta u_{c1}+\int_{l_{2}}^{l_{2}}\left(C_{33E}Adx\right)\cdot \frac{\partial^{2}w_{E2}}{\partial z^{2}}\cdot \delta u_{E2}+\int_{l_{2}}^{l_{3}}\left(E_{0}Adx\right)\cdot \frac{\partial^{2}w_{p3}}{\partial z^{2}}\cdot \delta u_{p3}\\ & +\int_{l_{3}}^{l_{4}}\left(C_{33E}Adx\right)\cdot \frac{\partial^{2}w_{E4}}{\partial z^{2}}\cdot \delta u_{E4}+\int_{l_{4}}^{l_{5}}\left(C_{33c}Adx\right)\cdot \frac{\partial^{2}w_{c5}}{\partial z^{2}}\cdot \delta u_{c5} \end{array}$$

By substituting Equations (9) and (25) into Equation (28), ${\delta W}_{E}$ can be obtained as:

(29) $$\delta W_{E}=-AT_{n}\left(t\right)\cdot \left(\lambda_{1n}C_{33c}B_{1}+\lambda_{2n}C_{33E}B_{2}+\lambda_{3n}E_{0}B_{3}+\lambda_{4n}C_{33E}B_{4}+\lambda_{5n}C_{33c}B_{5}\right)$$

In order to obtain the virtual work ${\delta W}_{\delta \left(t\right)}$ of the impact load  δ(t), by substituting $l_{5}$ into Equation (25b) to obtain the virtual displacement at the free end, ${\delta W}_{\delta \left(t\right)}\;$ is expressed as:

(30) $$\delta W_{\delta \left(t\right)}=\delta \left(t\right)A\left(a_{5n}\cos\sqrt{\lambda_{5n}}l_{5}+b_{5n}\sin\sqrt{\lambda_{5n}}l_{5}\right)$$

Summation of ${\delta W}_{i}$ , ${\delta W}_{E}$ and ${\delta W}_{\delta \left(t\right)}$ gives the total virtual work, equating it to zero:

(31) $$\delta W_{i}+\delta W_{E}+\delta W_{\delta \left(t\right)}=0$$

Substituting Equations (27), (29) and (30) into Equation (31), one obtains:

(32) $${\ddot{T}}_{n}(t)+\bar{\lambda_{n}}T_{n}(t)=\frac{\delta (t)}{M_{n}}\cdot (a_{5n}\cos\sqrt{\lambda_{5n}}l_{5}+b_{5n}\sin\sqrt{\lambda_{5n}}l_{5})$$

where $M_{n}{\;=\;\rho }_{c}B_{1}{\;+\;\rho }_{E}B_{2}{\;+\;\rho }_{p}B_{3}{\;+\;\rho }_{E}B_{4}{\;+\;\rho }_{c}B_{5}$ and the Dirac function δ(t) can be expressed as:

(33) $$\delta (t)=\{\begin{array}{c} \infty ,t=0\\ 0,t\neq 0 \end{array}$$

Writing the solution of the Equation (32) in the form of the Duhamel’s integral, one obtains:

(34) $$\begin{array}{cc} T_{n}(t) & =\frac{\left(a_{5n}\cos\sqrt{\lambda_{5n}}l_{5}+b_{5n}\sin\sqrt{\lambda_{5n}}l_{5}\right)}{M_{n}\sqrt{\bar{\lambda_{n}}}}\cdot \int_{0}^{t}\delta (\tau )\sin\sqrt{\bar{\lambda_{n}}}(t-\tau )d\tau \\ & =\frac{\left(a_{5n}\cos\sqrt{\lambda_{5n}}l_{5}+b_{5n}\sin\sqrt{\lambda_{5n}}l_{5}\right)}{M_{n}\sqrt{\bar{\lambda_{n}}}}\cdot \sin\sqrt{\bar{\lambda_{n}}}t \end{array}$$

Therefore, the accurate vibration analysis of the 2-2 cement-based piezoelectric composite sensor excited by the impact load can be obtained as:

Displacement functions:

(35) $$\{\begin{array}{c} w_{ci}(z,t)=\sum_{n=1}^{\infty }\frac{D_{n}}{\sqrt{\bar{\lambda_{n}}}}\cdot \sin\sqrt{\bar{\lambda_{n}}}t(a_{in}\cos\sqrt{\lambda_{in}}z+b_{in}\sin\sqrt{\lambda_{in}}z);\;t\geq 0,\;l_{i-1}\leq z\leq l_{i},\;i=1,5\\ w_{Ei}(z,t)=\sum_{n=1}^{\infty }\frac{D_{n}}{\sqrt{\bar{\lambda_{n}}}}\cdot \sin\sqrt{\bar{\lambda_{n}}}t(a_{in}\cos\sqrt{\lambda_{in}}z+b_{in}\sin\sqrt{\lambda_{in}}z);\;t\geq 0,\;l_{i-1}\leq z\leq l_{i},\;i=2,4\\ w_{pi}(z,t)=\sum_{n=1}^{\infty }\frac{D_{n}}{\sqrt{\bar{\lambda_{n}}}}\cdot \sin\sqrt{\bar{\lambda_{n}}}t(a_{in}\cos\sqrt{\lambda_{in}}z+b_{in}\sin\sqrt{\lambda_{in}}z);\;t\geq 0,\;l_{i-1}\leq z\leq l_{i},\;i=3 \end{array}$$

where $D_{n}{\;=\;(a}_{5n}\cos\sqrt{\lambda_{5n}}l_{5}{\;+\;b}_{5n}\sin\sqrt{\lambda_{5n}}l_{5})/M_{n}$ ;

Stress functions:

(36) $$\{\begin{array}{c} \sigma_{ci}(z,t)=\sum_{n=1}^{\infty }\frac{C_{33c}D_{n}\sqrt{\lambda_{in}}}{\sqrt{\bar{\lambda_{n}}}}\cdot \sin\sqrt{\bar{\lambda_{n}}}t(b_{in}\cos\sqrt{\lambda_{in}}z-a_{in}\sin\sqrt{\lambda_{in}}z);\;t\geq 0,\;l_{i-1}\leq z\leq l_{i},\;i=1,5\\ \sigma_{Ei}(z,t)=\sum_{n=1}^{\infty }\frac{C_{33E}D_{n}\sqrt{\lambda_{in}}}{\sqrt{\bar{\lambda_{n}}}}\cdot \sin\sqrt{\bar{\lambda_{n}}}t(b_{in}\cos\sqrt{\lambda_{in}}z-a_{in}\sin\sqrt{\lambda_{in}}z);\;t\geq 0,\;l_{i-1}\leq z\leq l_{i},\;i=2,4\\ \sigma_{pi}(z,t)=\sum_{n=1}^{\infty }\frac{E_{0}D_{n}\sqrt{\lambda_{in}}}{\sqrt{\bar{\lambda_{n}}}}\cdot \sin\sqrt{\bar{\lambda_{n}}}t(b_{in}\cos\sqrt{\lambda_{in}}z-a_{in}\sin\sqrt{\lambda_{in}}z);\;t\geq 0,\;l_{i-1}\leq z\leq l_{i},\;i=3 \end{array}$$

Strain functions:

(37) $$\{\begin{array}{c} \varepsilon_{ci}(z,t)=\sum_{n=1}^{\infty }\frac{D_{n}\sqrt{\lambda_{in}}}{\sqrt{\bar{\lambda_{n}}}}\cdot \sin\sqrt{\bar{\lambda_{n}}}t(b_{in}\cos\sqrt{\lambda_{in}}z-a_{in}\sin\sqrt{\lambda_{in}}z);\;t\geq 0,\;l_{i-1}\leq z\leq l_{i},\;i=1,5\\ \varepsilon_{Ei}(z,t)=\sum_{n=1}^{\infty }\frac{D_{n}\sqrt{\lambda_{in}}}{\sqrt{\bar{\lambda_{n}}}}\cdot \sin\sqrt{\bar{\lambda_{n}}}t(b_{in}\cos\sqrt{\lambda_{in}}z-a_{in}\sin\sqrt{\lambda_{in}}z);\;t\geq 0,\;l_{i-1}\leq z\leq l_{i},\;i=2,4\\ \varepsilon_{pi}(z,t)=\sum_{n=1}^{\infty }\frac{D_{n}\sqrt{\lambda_{in}}}{\sqrt{\bar{\lambda_{n}}}}\cdot \sin\sqrt{\bar{\lambda_{n}}}t(b_{in}\cos\sqrt{\lambda_{in}}z-a_{in}\sin\sqrt{\lambda_{in}}z);\;t\geq 0,\;l_{i-1}\leq z\leq l_{i},\;i=3 \end{array}$$

Velocity functions:

(38) $$\{\begin{array}{c} v_{ci}(z,t)=\sum_{n=1}^{\infty }D_{n}\cdot \cos\sqrt{\bar{\lambda_{n}}}t(a_{in}\cos\sqrt{\lambda_{in}}z+b_{in}\sin\sqrt{\lambda_{in}}z);\;t\geq 0,\;l_{i-1}\leq z\leq l_{i},\;i=1,5\\ v_{Ei}(z,t)=\sum_{n=1}^{\infty }D_{n}\cdot \cos\sqrt{\bar{\lambda_{n}}}t(a_{in}\cos\sqrt{\lambda_{in}}z+b_{in}\sin\sqrt{\lambda_{in}}z);\;t\geq 0,\;l_{i-1}\leq z\leq l_{i},\;i=2,4\\ v_{pi}(z,t)=\sum_{n=1}^{\infty }D_{n}\cdot \cos\sqrt{\bar{\lambda_{n}}}t(a_{in}\cos\sqrt{\lambda_{in}}z+b_{in}\sin\sqrt{\lambda_{in}}z);\;t\geq 0,\;l_{i-1}\leq z\leq l_{i},\;i=3 \end{array}$$

Acceleration functions:

(39) $$\{\begin{array}{c} a_{ci}(z,t)=-\sum_{n=1}^{\infty }D_{n}\sqrt{\bar{\lambda_{n}}}\cdot \sin\sqrt{\bar{\lambda_{n}}}t(a_{in}\cos\sqrt{\lambda_{in}}z+b_{in}\sin\sqrt{\lambda_{in}}z);\;t\geq 0,\;l_{i-1}\leq z\leq l_{i},\;i=1,5\\ a_{Ei}(z,t)=-\sum_{n=1}^{\infty }D_{n}\sqrt{\bar{\lambda_{n}}}\cdot \sin\sqrt{\bar{\lambda_{n}}}t(a_{in}\cos\sqrt{\lambda_{in}}z+b_{in}\sin\sqrt{\lambda_{in}}z);\;t\geq 0,\;l_{i-1}\leq z\leq l_{i},\;i=2,4\\ a_{pi}(z,t)=-\sum_{n=1}^{\infty }D_{n}\sqrt{\bar{\lambda_{n}}}\cdot \sin\sqrt{\bar{\lambda_{n}}}t(a_{in}\cos\sqrt{\lambda_{in}}z+b_{in}\sin\sqrt{\lambda_{in}}z);\;t\geq 0,\;l_{i-1}\leq z\leq l_{i},\;i=3 \end{array}$$

The electric potential of the piezoelectric layer:

(40) $$\phi (z,t)=\sum_{n=1}^{\infty }\frac{e_{33}D_{n}}{\varepsilon_{33}^{S}\sqrt{\bar{\lambda_{n}}}}\cdot \sin\sqrt{\bar{\lambda_{n}}}t[a_{3n}(\cos\sqrt{\lambda_{3n}}z-\cos\sqrt{\lambda_{3n}}l_{2})+b_{3n}(\sin\sqrt{\lambda_{3n}}z-\sin\sqrt{\lambda_{3n}}l_{2})];\;t\geq 0,\;l_{2}\leq z\leq l_{3}$$

Electric field intensity of piezoelectric layer:

(41) $$E(z,t)=\sum_{n=1}^{\infty }-\frac{e_{33}D_{n}\sqrt{\lambda_{3n}}}{\varepsilon_{33}^{S}\sqrt{\bar{\lambda_{n}}}}\cdot \sin\sqrt{\bar{\lambda_{n}}}t(b_{3n}\cos\sqrt{\lambda_{3n}}z-a_{3n}\sin\sqrt{\lambda_{3n}}z);\;t\geq 0,\;l_{2}\leq z\leq l_{3}$$

Till now, all the mechanical and electrical solutions have been obtained by using the mode summation method and the principle of virtual work.

## 4. Comparisons and Discussions

In this section, by comparing the theoretical and the numerical solutions, the modal interception of the mode summation method, and the critical impact load value in the numerical simulation are discussed. We also analyze the influence of the material and geometrical parameters on the mechanical and electrical behaviors of the sensor.

### 4.1. Comparions between the Theoretical Solutions and the Numerical Solutions

**Figure 2:**
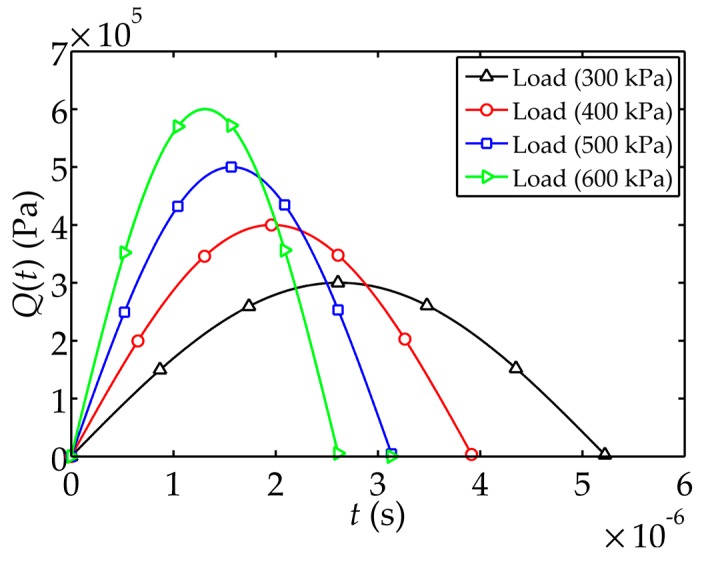
Schematics of the transient haversine wave load Q(t) .

The thickness of the sensor is taken as 0.02 m. The material properties used in Li’s experiments [22] are adopted and are listed in Table 1. ANSYS is used for the numerical simulation, where an analytical model of the size 0.001 m × 0.001 m × 0.02 m is considered. The piezoelectric layer is defined as a Solid5 unit, cement layers and electrode layers are both defined as Solid45 units. The interlayer contact is glue. The unit partition of the model is divided into five segments along the *x*-axis and *y*-axis, and 100 segments along the *z*-axis by using the free meshing method. The upper and lower surfaces of the piezoelectric layer in the *z*-axis direction are subjected to the piezoelectric coupling. The electric potential of the lower surface of the piezoelectric layer is set to zero. The model is loaded and solved after the symmetrical boundary conditions are set on the four sides of the model. The impact load Q(t)  used in the numerical simulation has the form of a transient haversine wave and is shown in Figure 2. The relation $\int_{-\infty }^{+\infty }Q(t)dt\;=\;1$ holds. The theoretical solutions are worked out by using common programming language with only about 6s. The calculation time of ANSYS simulation is 13 min.

**Figure 3:**
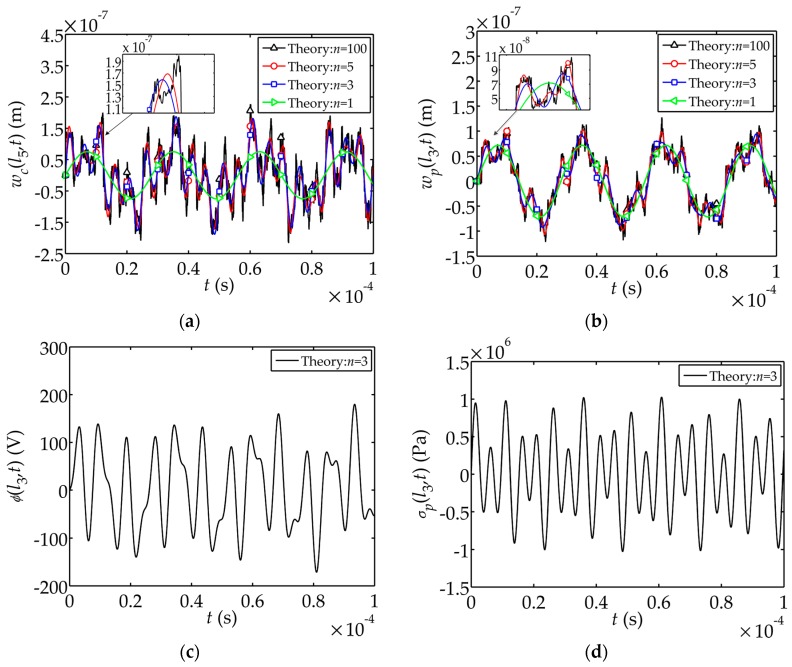
Theoretical solution of displacement, electric potential and stress: (**a**) Displacement $w_{c}{(l}_{5},t)$ ; (**b**) Displacement $w_{p}{(l}_{3},t)$ ; (**c**) Electric potential $\phi {(l}_{3},t)$ ; (**d**) Stress $\sigma_{p}{(l}_{3},t)$ .

Figure 3a,b show the influences of the number of modes *n* involved in the summation on the displacement. It is found that the participation of the high-order modes would cause slight increase in the displacement amplitude and longer vibration duration. There are not much dramatic changes in the modal curves when high-order modes join in. The theoretical solutions of the electric potential and stress are plotted in Figure 3c,d, where *n*  = 3 .

**Figure 4:**
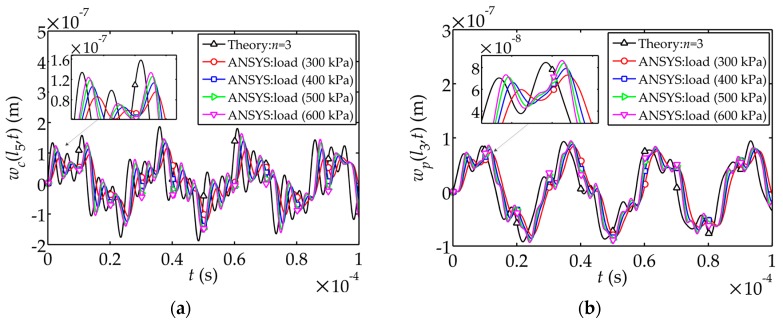

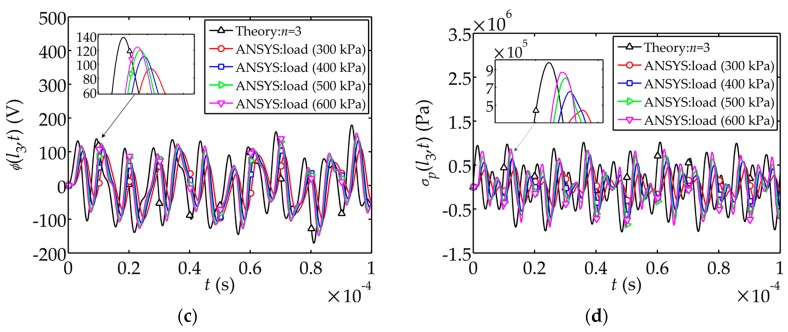
Comparisons of theoretical solutions and the numerical solutions of displacement, electric potential and stress: (**a**) Displacement $w_{c}{(l}_{5},t)$ ; (**b**) Displacement $w_{p}{(l}_{3},t)$ ; (**c**) Electric potential $\phi {(l}_{3},t)$ ; (**d**) Stress $\sigma_{p}{(l}_{3},t)$ .

In order to obtain the critical impact load value in the numerical simulation, comparisons between theoretical solutions and the numerical solutions with different peak value of the impact load Q(t)  are shown in Figure 4. The peak value of Q(t) takes 300 kPa, 400 kPa, 500 kPa and 600 kPa respectively. It is observed that the numerical solution is closer to the theoretical solution as the peak value of Q(t) becomes larger. In particular, the electric potential amplitude in the simulation with 300 kPa peak value is approximately 50 V less than the theoretical amplitude, and the stress amplitude with 400 kPa peak value is less about 400 kPa than the theoretical amplitude (see Figure 4c,d). Therefore, it is appropriate to use the impact load Q(t) with 500 kPa or larger peak value in the numerical simulation.

### 4.2. Material and Geometrical Parameters of Composite Properties

It is assumed that three types of elastic electrode layers are made of H62-brass, aluminum and (gold-tin, 80% wt % Au-20 wt % Sn), respectively. The material constants of H62-brass [17], aluminum [23] and (gold-tin, 80% wt % Au-20 wt % Sn) [24] are listed in Table 2.

**Figure 5:**
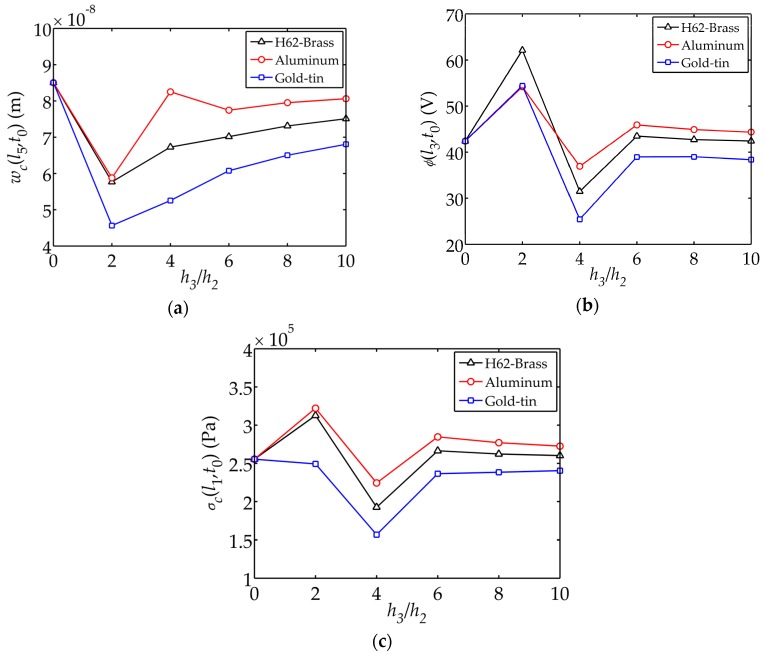
Displacement $w_{c}{(l}_{5},t_{0})$ , electric potential $\phi {(l}_{3},t_{0})$ and stress $\sigma_{c}{(l}_{1},t_{0})$ changing with versus the thickness ratio $h_{3}{/h}_{2}$ : (**a**) Displacement $w_{c}{(l}_{5},t_{0})$ ; (**b**) Electric potential $\phi {(l}_{3},t_{0})$ ; (**c**) Stress $\sigma_{c}{(l}_{1},t_{0})$ .

Figure 5 demonstrates the displacement $w_{c}{(l}_{5},t_{0})$ , electric potential $\phi {(l}_{3},t_{0})\;$ and stress $\sigma_{c}{(l}_{3},t_{0})$ versus the thickness ratio $h_{3}{/h}_{2}$ . Here $t_{0}\;=\;{0.70\;\times \;10}^{-5}\;$ s, the first displacement amplitude appeared at $t_{0}$ (shown in Figure 2a). $h_{3}$ and $h_{2}$ are the thickness of the piezoelectric layer and electrode layer, respectively. Meanwhile, the influence of the elastic electrode material on the displacement, electric potential and stress are also shown. By keeping the total thickness $l_{5}$ and the thickness of piezoelectric layer $h_{3}\;$ constant, it is observed that when $h_{3}/h_{2}\;=\;4$ , the internal stresses and electric potential are minimal. The tip displacement is larger with a thinner electrode layer. This is an indication that the mechanical and electrical characteristics can be enhanced by tailoring the geometry of the sensor. The impact of aluminum on the mechanical and electrical behaviors are relatively larger.

**Figure 6:**
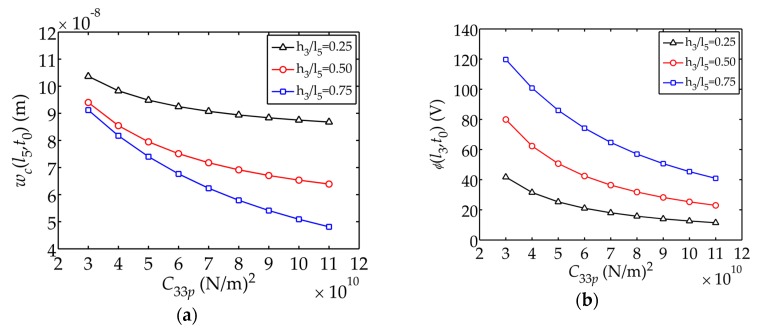

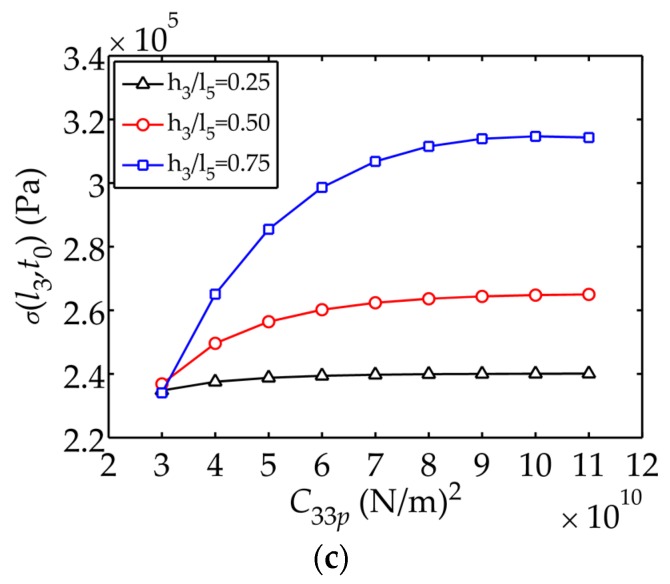
Displacement $w_{c}{(l}_{5},t_{0})$ , electric potential $\phi {(l}_{3},t_{0})$ and stress $\sigma_{c}{(l}_{1},t_{0})$ changing with $C_{33p}$ : (**a**) Displacement $w_{c}{(l}_{5},t_{0})$ ; (**b**) Electric potential $\phi {(l}_{3},t_{0})$ ; (**c**) Stress $\sigma_{c}{(l}_{1},t_{0})$ .

Figure 6 shows the displacement $w_{c}{(l}_{5},t_{0})$ , electric potential $\phi {(l}_{3},t_{0})$ and stress $\sigma_{c}{(l}_{1},t_{0})$ as functions of $C_{33p}$ respectively. The tip displacement and electric potential both decrease, while the internal stress increases, as $C_{33p}$ grows. It can be concluded that a sensor with smaller value of $C_{33p}$ can provide larger displacement and electric potential, therefore further causing smaller internal stress.

**Figure 7:**
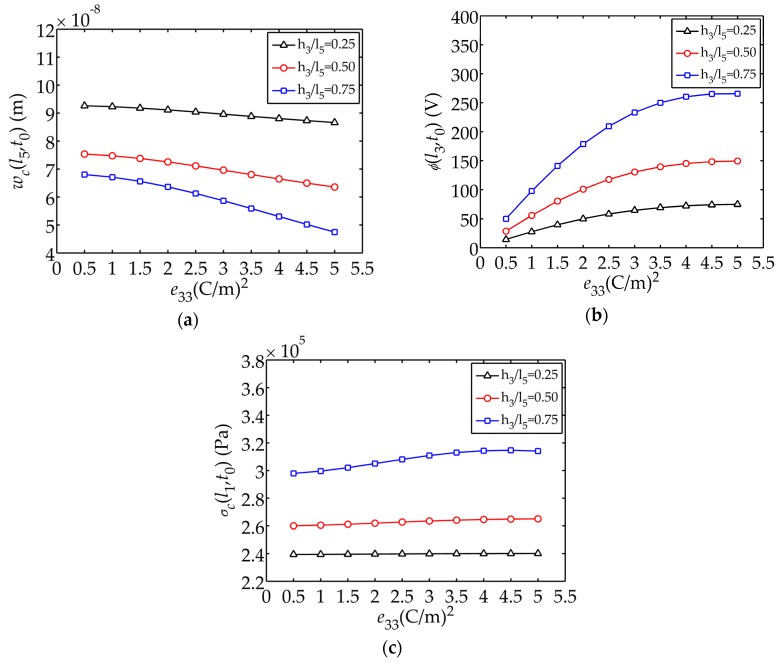
Displacement $w_{c}{(l}_{5},t_{0})$ , electric potential $\phi {(l}_{3},t_{0})$ and stress $\sigma_{c}{(l}_{1},t_{0})$ changing with $e_{33}$ : (**a**) Displacement $w_{c}{(l}_{5},t_{0})$ ; (**b**) Electric potential $\phi {(l}_{3},t_{0})$ ; (**c**) Stress $\sigma_{c}{(l}_{1},t_{0})$ .

Figure 7 illustrates the displacement $w_{c}{(l}_{5},t_{0})$ , electric potential $\phi {(l}_{3},t_{0})$ and stress $\sigma_{c}{(l}_{1},t_{0})$ as functions of $e_{33}$ respectively. It can be easily seen that the influence of $e_{33}$ on the electric potential is more obvious. The larger $e_{33}$ is, the larger the electric potential generated is.

**Figure 8:**
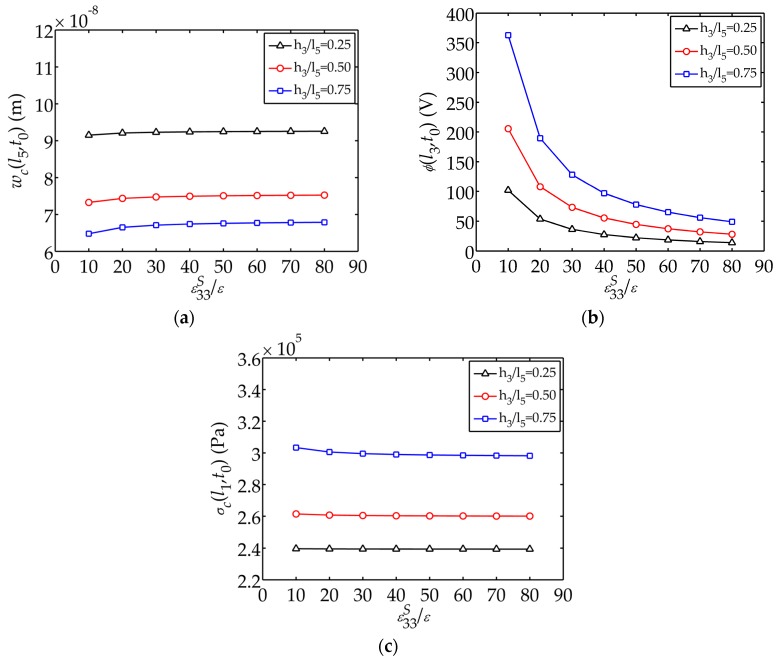
Displacement $w_{c}{(l}_{5},t_{0})$ , electric potential $\phi {(l}_{3},t_{0})$ and stress $\sigma_{c}{(l}_{1},t_{0})$ changing with $\;\varepsilon_{33}^{S}/\varepsilon$ : (**a**) Displacement $w_{c}{(l}_{5},t_{0})$ ; (**b**) Electric potential $\phi {(l}_{3},t_{0})$ ; (**c**) Stress $\sigma_{c}{(l}_{1},t_{0})$ .

Figure 8 plots the displacement $w_{c}{(l}_{5},t_{0})$ , electric potential $\phi {(l}_{3},t_{0})$ and stress $\sigma_{c}{(l}_{1},t_{0})$ as functions of $\varepsilon_{33}^{S}/\varepsilon$ , respectively. It can be described that the electric potential decreases as $\varepsilon_{33}^{S}/\varepsilon$ increases and the trend flattens when $\varepsilon_{33}^{S}/\varepsilon$ is larger than 50. Figure 6, Figure 7 and Figure 8 also reveal the influence of the thickness ratio $h_{3}/l_{5}$ on the mechanical and electrical behaviors. Composites with thicker piezoelectric layers can generate larger electric potential as well as larger internal stresses and smaller tip displacements. A composite with a thinner piezoelectric layer has better mechanical behaviors. $h_{3}/l_{5}$ of 0.50 is a relatively good geometrical parameters for the sensor.

**Figure 9:**
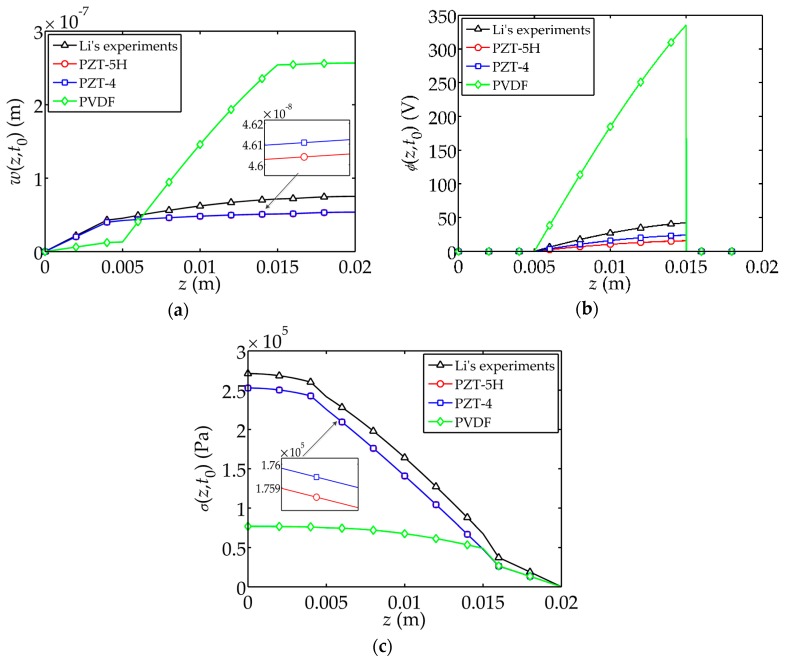
Influences of piezoelectric materials on the dynamic behaviors of the sensor. (**a**) Displacement $w(z,t_{0})$ ; (**b**) Electric potential $\phi (z,t_{0})$ ; (**c**) Stress $\sigma (z,t_{0})$ .

The influence of the piezoelectric material on the displacement $w(z,t_{0})$ electric potential $\phi (z,t_{0})$ stress $\sigma (z,t_{0})$ are shown in Figure 9. PZT-5H, PZT-4 and PVDF [25] are discussed as common piezoelectric materials, and a comparison with Li’s experiments [22] is presented. The material constants of PZT-5H, PZT-4 and PVDF are listed in Table 3.

For PVDF, obvious differences in mechanical and electrical behaviors are observed as compared to the case for PZT, as shown in Figure 9. *z* is the direction along the thickness direction of the composite. The tip displacement and electric potential are large enough for the sensor under impact load. Meanwhile, the resulting stress is relatively small compared with PZT.

**Figure 10:**
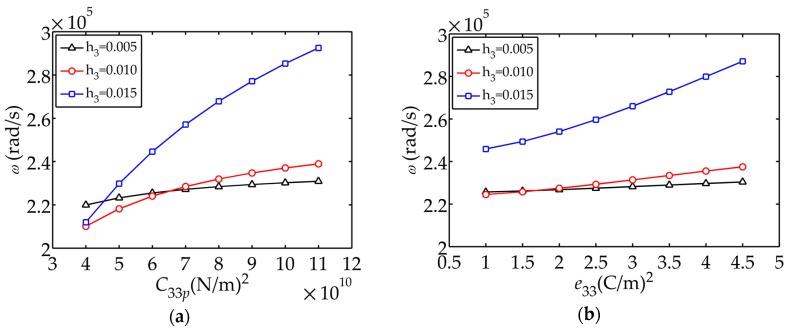

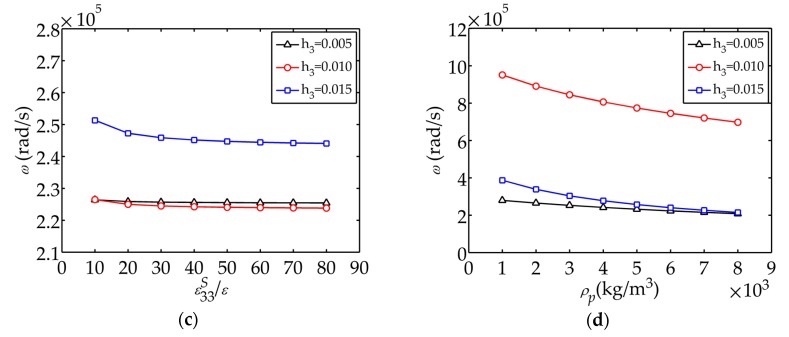
Influence of material parameters on ω : (**a**) $C_{33p}$ ; (**b**) $e_{33}$ ; (**c**) $\varepsilon_{33}^{S}{/\varepsilon }_{0}$ ; (**d**) ${\;\rho }_{p}$ .

Figure 10 demonstrates the influence of material parameters on the fundamental frequency. It can be seen that the fundamental frequency ω increases as $C_{33p},$ $e_{33}$ and $\varepsilon_{33}^{S}/\varepsilon$ grow, decreases as $\rho_{p}$ increases. Figure 10 also shows that the thicker the piezoelectric layer, the greater the effect of the piezoelectric coefficients on the frequency (larger rate of change).

## 5. Conclusions

Based on the theory of piezo-elasticity, an accurate mechanical and electrical analysis of the 2-2 cement-based piezoelectric sensor are presented in this paper. Theoretical solutions are obtained with the mode summation method and the principle virtual work. Through comparisons with numerical solutions, the following conclusions can be drawn:

(1) For theoretical solutions, the vibration modal curves of the sensor subjected to the impact load have no obvious change after the addition of high-order modes. It’s sufficient to analyze and summate the first three modes. Numerical simulations have good agreement with the theoretical solutions when the peak value of impact load Q(t) is larger than 500 kPa.

(2) By keeping the total thickness of the sensor and the thickness of piezoelectric layer $h_{3}$ constant, the sensor shows good mechanical properties with a thickness ratio $h_{3}/h_{2}\;=\;4$ and good electrical property with a thickness ratio $h_{3}/h_{2}\;=\;2$ . Aluminum as the elastic electrode material has a relatively large impact on the tip displacement, electric potential and internal stress.

(3) Through adjusting the thickness of layers and material parameters, the displacement, electric potential and stress of the sensor could be optimized. The coefficient $C_{33p}$ has obvious influence on $w_{c}{(l}_{5},t_{0}),$ $\sigma_{c}{(l}_{1},t_{0})$ and $\phi {(l}_{3}{,t}_{0})$ ; while $e_{33}$ and ${\;\varepsilon }_{33}^{S}/\varepsilon$ have larger influence on $\phi {(l}_{3},t_{0})$ . For the sensor, smaller $C_{33p}$ and $\varepsilon_{33}^{S}/\varepsilon$ , larger $e_{33}$ would provide better mechanical and electrical behaviors. PVDF as piezoelectric material can provide stronger electric power as well as causing smaller internal stress.

(4) The frequency of the composite could also be controlled by choosing different materials and tailoring the geometry of the composite. The thicker the piezoelectric layer, the greater the effect of the piezoelectric coefficients on the frequency (lager changing rate).

By analyzing the dynamic characteristics of the sensor, the present work would provide certain guidance for the sensor structure design, material selection and impact load design, both in simulations and experiments.

## Figures and Tables

**Table 1:** Properties of piezoelectric ceramics, elastic electrode and cement.

Material	Thickness	Density	Elastic Stiffness Coefficient	Poisson’s Ratio	Piezoelectric Coefficient	Permittivity Coefficient
Ordinary Portland Cement	0.004 m	${2500\;\mathrm{kg}/m}^{3}$	${2.5\times 10}^{10}\;\mathrm{Pa}$	0.2	/	/
Piezoelectric Ceramics	0.010 m	${5700\;\mathrm{kg}/m}^{3}$	${6.0\times 10}^{10}\;\mathrm{Pa}$	/	${0.75\;C/m}^{2}$	${52.5\varepsilon }_{0}\;$ ^1^
H62-Brass	0.001 m	${8430\;\mathrm{kg}/m}^{3}$	${10.0\times 10}^{10}\;\mathrm{Pa}$	0.34	/	/

^1^ $\varepsilon_{0\;}=\;{8.85\;\times \;10\;}^{-12}\;F/m$ is the vacuum dielectric constant.

**Table 2:** Properties of elastic electrode.

Material	Density	Elastic Stiffness Coefficient
H62-Brass	${8430\;\mathrm{kg}/m}^{3}$	${10\times 10}^{10}\;\mathrm{Pa}$
Aluminum	${2536\;\mathrm{kg}/m}^{3}$	${7.0\times 10}^{10}\;\mathrm{Pa}$
Gold-tin 80% wt % Au 20 wt % Sn	${16,900\;\mathrm{kg}/m}^{3}$	${13.73\times 10}^{10}\;\mathrm{Pa}$

**Table 3:** Properties of piezoelectric materials.

Material	Density	Elastic Stiffness Coefficient	Piezoelectric Coefficient	Permittivity Coefficient
PZT-5H	${7500\;\mathrm{kg}/m}^{3}$	${11.7\times 10}^{10}\;\mathrm{Pa}$	${23.3\;C/m}^{2}$	${1470\varepsilon }_{0}\;$ ^1^
PZT-4	${7500\;\mathrm{kg}/m}^{3}$	${11.5\times 10}^{10}\;\mathrm{Pa}$	${15.1\;C/m}^{2}$	${635\varepsilon }_{0}\;$ ^1^
PVDF	${1780\;\mathrm{kg}/m}^{3}$	${0.25\times 10}^{10}\;\mathrm{Pa}$	${0.16\;C/m}^{2}$	${13\varepsilon }_{0}\;$ ^1^

^1^ $\varepsilon_{0}\;=\;{8.85\;\times \;10\;}^{-12}\;F/m$ is the vacuum dielectric constant.
